# Effects of Cheonggukjang (Fermented Soybean) on the Development of Colitis-Associated Colorectal Cancer in Mice

**DOI:** 10.3390/foods12020383

**Published:** 2023-01-13

**Authors:** Hyeon-Ji Lim, In-Sun Park, Su-Ji Jeong, Gwang-Su Ha, Hee-Jong Yang, Do-Youn Jeong, Seon-Young Kim, Chan-Hun Jung

**Affiliations:** 1 Jeonju AgroBio-Materials Institute, Wonjangdong-gil 111-27, Jeonju 54810, Republic of Korea; 2 Microbial Institute for Fermentation Industry, Sunchang 56048, Republic of Korea

**Keywords:** colorectal cancer, chronic inflammation, inflammatory bowel disease, cheonggukjang, anti-cancer effects, functional food

## Abstract

Colorectal cancer (CRC) is the third most common type of cancer and is caused by multiple factors. Chronic inflammation, known to cause inflammatory bowel disease (IBD), is closely associated with CRC. Cheonggukjang (CJ), a traditional Korean fermented soybean, is a functional food with anti-inflammatory effects in the intestines, but its anti-cancer effects have not yet been explored. In this study, we investigated the cancer-protective effects of cheonggukjang in an azoxymethane/DSS (AOM/DSS)-induced colitis-associated colorectal cancer (CAC) mouse model. The CJ alleviated AOM/DSS-induced pathological symptoms such as colonic shortening, increased spleen weight, tumor formation, and histological changes. It also modulated pro-inflammatory and anti-inflammatory cytokine levels via the suppression of NF-κB and inflammatory mediator signaling pathways. Furthermore, the CJ improved intestinal integrity by regulating mucin-associated and tight junction proteins. In addition, it suppressed tumor growth by regulating apoptosis and proliferation. These results highlight the anti-tumor effects of CJ in an AOM/DSS-induced CAC mouse model.

## 1. Introduction

Colorectal cancer (CRC) is the third most common type of cancer in both men and women worldwide [1]. In 2018, the overall CRC incidence rate in Korea was 11.4%, 12.9% in men and 9.8% in women, with a mortality rate of 10% in men and 12.6% in women [2]. Chronic inflammation leads to various organ-specific diseases, depending on where the inflammation occurs [3]. In particular, chronic inflammation in the intestine causes inflammatory bowel disease (IBD), which increases the risk of developing CRC, also known as colitis-associated colorectal cancer (CAC) [4].

IBD is associated with multiple genetic, microbial, environmental, and immune-mediated factors [5,6]. However, the accurate etiology of IBD is still unknown. Recent studies have indicated that pro-inflammatory cytokines and gut dysbiosis induce both intestinal inflammation and the disruption of normal mucosal immunity, resulting in IBD [7,8]. Consequently, the suppression of intestinal inflammation may help prevent inflammation-associated colon cancer [9].

Cheonggukjang (CJ) is a soybean paste made through the fermentation of boiled soybeans. It is rich in substances that exhibit various biological activities, such as poly γ-glutamic acid (γ-PGA), isoflavone, saponins, phenolic acids, and flavonoids [10]. In many studies, CJ has emerged as a functional food with anti-obesity, antioxidant, and anti-inflammatory effects in bowel disease [10,11,12]. We have previously shown that CJ contains beneficial probiotics and is effective in preventing inflammatory diseases by suppressing the inflammatory signaling pathway in dextran sodium sulfate (DSS)-induced colitis mice [13]. However, to the best of our knowledge, the preventive effect of CJ on inflammatory colorectal tumorigenesis has not been elucidated. In this study, we report the anti-tumor growth effects of CJ by inhibiting azoxymethane (AOM)/DSS-induced pathological symptoms in an AOM/DSS-induced CAC mouse model.

## 2. Materials and Methods

### 2.1. Preparation of the CJ

The CJ was obtained from the Microbial Institute for Fermentation Industry (Sunchang-gun, Jeollabuk-do, Republic of Korea) as described previously [13]. It was produced using the traditional method of Kangjin-gun (Jeollanam-do, Republic of Korea) and had a moisture content of 53.15%. The CJ was dissolved in distilled water at 500 mg/kg and then stored at −20 °C before being orally administered to mice.

### 2.2. AOM/DSS-Induced Colorectal Cancer Model and CJ Treatment

A total of 32 male BALB/c mice (five-week-old) were purchased from Damool Science (Daejeon, Republic of Korea). The mice were maintained at 20–24 °C with a 12 h light/dark cycle and a relative humidity of 50–60%. After 1 week of acclimatization, the mice were divided into four groups (*n* = 8): NOR (normal group; only water), CON (control group; AOM (10 mg/kg)/DSS (2%)), PC (positive control group; AOM (10 mg/kg)/DSS (2%) and 5-aminosalicylic acid (75 mg/kg/day, 5-ASA, Sigma-Aldrich, St. Louis, MO, USA)), and CJ (CJ treated group; AOM (10 mg/kg)/DSS (2%) and CJ (100 mg/kg/day)). AOM/DSS-induced CAC was initiated by injecting the mice intraperitoneally with AOM (10 mg/kg, Sigma-Aldrich, St. Louis, MO, USA) on day 0. After 1 week, the mice were administered DSS (2%, MP Biomedicals, Irvine, CA, USA) for another week, followed by water for 2 weeks for recovery. This treatment was repeated twice (days 7–14, 32–39). All experimental animal procedures were performed within the guidelines of and approved by the Jeonju AgroBio-Materials Institute’s Animal Care and Use Committee (JAMI IACUC 2022004).

### 2.3. ELISA Analysis

The serum levels of TNF-α (MTA00B), IFN-γ (MIF00), IL-6 (M6000B), IL-1β (MLB00C), IL-4 (M4000B), and IL-10 (M1000B) were analyzed using commercial ELISA kits (R&D Systems, Minneapolis, MN, USA) according to the manufacturer’s recommendations.

### 2.4. Quantitative Real-Time PCR (qRT-PCR) Analysis

Total RNA was extracted from colonic tissues using a Hybrid-R^TM^ kit (GeneAll, Seoul, Republic of Korea). First, cDNA was synthesized with the BioFACT^TM^ 2X RT Pre-Mix (BIOFACT, Daejeon, Republic of Korea). Next, qRT-PCR was undertaken using the BioFACT^TM^ 2X Real-Time PCR Master Mix (Bio-Fact) and analyzed with a sequence detection system (CFX96, Bio-Rad, Hercules, CA, USA). The primer sequences used are listed in Table 1.

### 2.5. Western Blotting

Western blotting was performed as described previously [13]. Briefly, about 20 μg of cell lysate was separated using a 10% SDS-PAGE and transferred to a PVDF membrane using the Trans-Blot Turbo Transfer system (Bio-Rad). The membranes were probed with specific antibodies: anti-iNOS, anti-COX-2, anti-p-p65, anti-p65, anti-Bax, anti-p53, anti-Bcl-2, anti-Bcl-X_L_, and anti-β-actin (Cell Signaling Technology, Danvers, MA, USA). The protein–antibody binding was visualized using the enhanced chemiluminescence (ECL) detection system (Amersham Imager 600, GE Healthcare, Chicago, IL, USA).

### 2.6. Hematoxylin & Eosin (H&E) Staining and Immunohistochemistry

Colon tissue sections (4 μm thick) were stained with H&E. For immunolabeling, the sections were incubated with specific antibodies (anti-Muc2, anti-ZO-1, anti-occludin, anti-proliferating cell nuclear antigen (PCNA; Cell Signaling Technology, Danvers, MA, USA), and anti-Ki-67 (Abcam, Boston, MA, USA)) overnight at 4 °C in the dark. Next, the sections were incubated with either mouse or rabbit Envision plus polymer reagent (Dako, Glostrup Kommune, Denmark) for 30 min at 4 °C, and binding was detected by 3, 3’-diaminobenzidine (DAB) staining. Images were captured using a tissue slide scanner (Motic; Xiamen, China).

### 2.7. Statistical Analysis

A comparison of the statistical significance between groups was determined using a one-way ANOVA and a Tukey post-hoc test in GraphPad Prism (version 5.0; GraphPad Software, Inc., San Diego, CA, USA). The data are presented as mean ± standard deviation (SD). The statistical significance was set at *p* < 0.05.

## 3. Results and Discussion

### 3.1. CJ Attenuates Pathological Symptoms in Mice with AOM/DSS-Induced CAC

**Figure 1:**
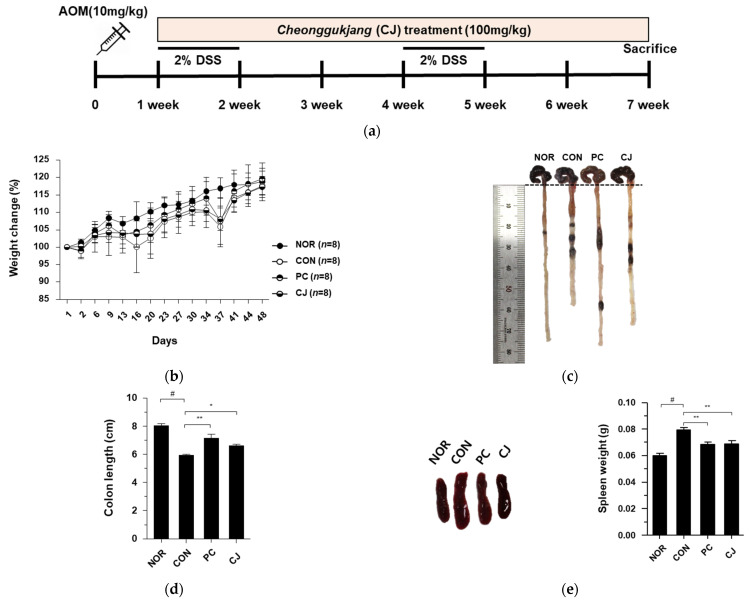
Effects of CJ on pathological symptoms of AOM/DSS-induced colorectal cancer (CAC) mice. (**a**) Experimental design; (**b**) weight change (%); (**c**) representative pictures of colon in mice; (**d**) colon length in mice; (**e**) representative pictures of spleen and spleen weight in mice. Values are means ± standard deviation (*n* = 8); NOR, normal group; CON, control group; PC, positive control group; CJ, cheonggukjang treated group; #, *p* < 0.05 versus normal group; **, *p* < 0.005; *, *p* < 0.05 versus control group.

Chronic inflammation, one of the major contributing factors in the development of IBD, is closely associated with CRC and can be prevented or delayed with anti-inflammatory agents [14,15]. We have previously studied the anti-inflammatory effects of CJ [13] and now aimed to determine the anti-cancer effects of CJ in CRC, which is closely related to chronic inflammation. Therefore, in this study, we investigated the anti-cancer effects of CJ using an AOM/DSS-induced CAC mouse model (Figure 1a). The administration of a 2% DSS reduced the body weights of AOM/DSS-treated mice. However, the lost body weight was regained when the DSS administration was discontinued (Figure 1b). The decrease in body weight was moderate in the PC (5-ASA treated) and CJ groups. Based on previous reports of reduced colon length in mice with AOM/DSS-induced CAC [16], we measured the colon lengths of the treated mice to evaluate the protective effects of CJ. Compared with the NOR group, the AOM/DSS-treated group (CON) had significantly shorter colons. However, the administration of 5-ASA and CJ restored the colon length (Figure 1c,d). Next, we measured the spleen weights, an indicator of the severity of tumor progression, of AOM/DSS-induced CAC mice [16]. The CON group showed an increase in spleen weight, which was reversed by the administration of 5-ASA and CJ (Figure 1e), supporting the anti-tumor effects of CJ. These findings indicate that CJ attenuates the pathological symptoms in mice with AOM/DSS-induced CAC.

### 3.2. CJ Suppresses Tumorigenesis in AOM/DSS-Induced CAC Mouse Model

**Figure 2:**
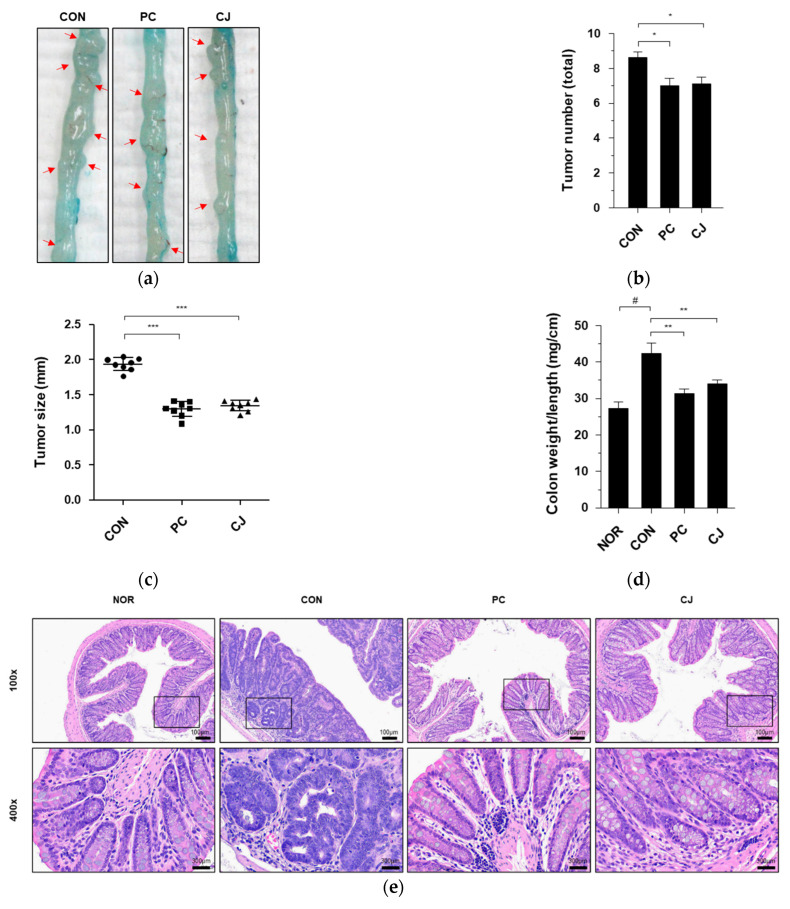
Effects of CJ on tumorigenesis in mice with AOM/DSS-induced CAC. (**a**) Representative pictures of colon tissue in the different groups of mice. Arrow indicates a tumor. Shown are the (**b**) total number of tumors, (**c**) tumor size, and (**d**) ratio of colon weight-to-colon length in the different groups of mice. All values are mean ± standard deviation (*n* = 8). (**e**) Representative histology for H&E staining of colon tissue. Magnification, 100× and 400×; Scale bar; 100 μm and 300 μm; NOR, normal group; CON, control group; PC, positive control group; CJ, cheonggukjang treated group. #, *p* < 0.05 versus normal group; ***, *p* < 0.001; **, *p* < 0.005; *, *p* < 0.05 versus control group.

AOM/DSS-induced CAC in mice is characterized by severe colitis with weight loss and a reduction in colon length, followed by the development of multiple colon tumors [17]. Therefore, we investigated the effects of CJ on intestinal tumorigenesis in mice with AOM/DSS-induced CAC. The AOM/DSS-treated mice showed an increase in macroscopic tumors, which was reversed following the administration of 5-ASA and CJ (Figure 2a.). The increased number of tumors in the CON group was reduced following the administration of 5-ASA and CJ (Figure 2b). Moreover, a significant difference was seen in the mean tumor size between the CON group and the PC and CJ groups (Figure 2c). Tumor formation and the thickening of the mucous membrane have been reported to increase the ratio of colon weight to colon length [18]. Therefore, we calculated the ratio of colon weight to colon length to confirm the inhibitory effects of CJ on tumorigenesis. As shown in Figure 2d, the weight-to-length ratio was significantly higher in the CON group than in the NOR group. However, this increase was significantly attenuated by the administration of 5-ASA and CJ (Figure 2d). H&E staining is used for the histological diagnosis of various diseases, including IBD and cancer [19,20]. The pathological features of AOM/DSS-induced CAC in mice include inflammatory cell infiltration, crypt abscesses, and hyperchromatic nuclei [17,21]. The histopathological features related to CRC development were examined with H&E staining. Inflammatory cell infiltration and hyperchromatic nuclei were observed in the colon tissue of the CON group, and they were significantly ameliorated by the administration of PC and CJ (Figure 2e). These results indicate that CJ suppresses AOM/DSS-induced colitis-associated tumorigenesis in mice.

### 3.3. CJ Modulates the Expression of Inflammatory Cytokines in AOM/DSS-Induced CAC Mice

**Figure 3:**
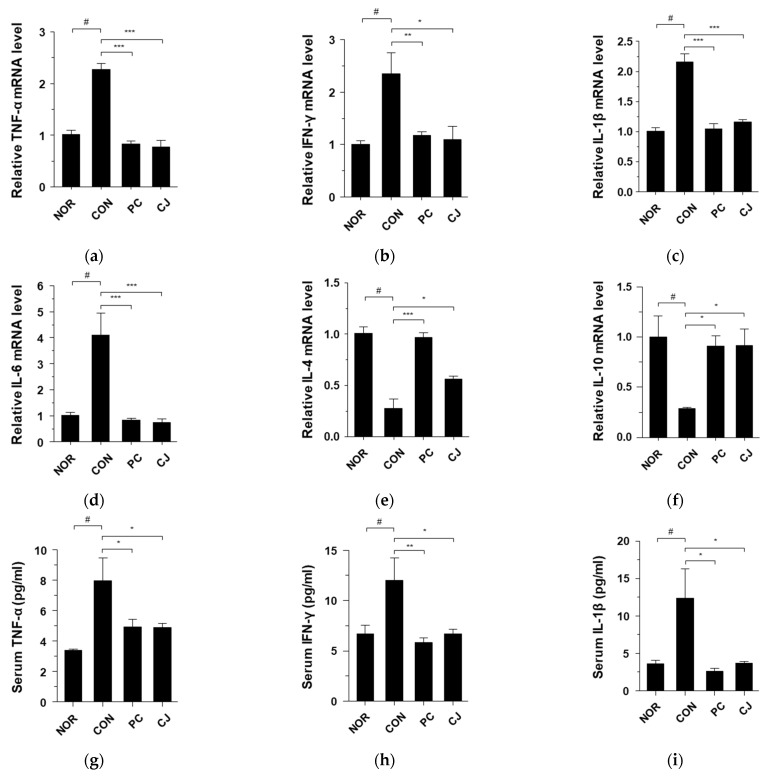

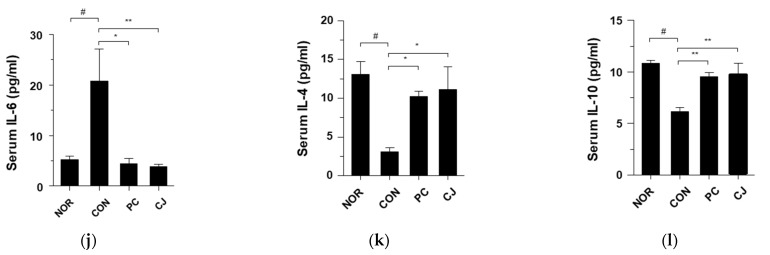
Effects of CJ on the expression of inflammatory cytokines in mice with AOM/DSS-induced CAC. Shown are the mRNA levels of (**a**) TNF-α, (**b**) IFN-γ, (**c**) IL-1β, (**d**) IL-6, (**e**) IL-4, and (**f**) IL-10 in the colon and protein levels of (**g**) TNF-α, (**h**) IFN-γ, (**i**), IL-1β, (**j**) IL-6, (**k**) IL-4, and (**l**) IL-10 in the serum. Values are mean ± standard deviation (*n* = 8); NOR, normal group; CON, control group; PC, positive control group; CJ, cheonggukjang treated group. #, *p* < 0.05 versus normal group; ***, *p* < 0.001; **, *p* < 0.005; *, *p* < 0.05 versus control group.

AOM/DSS-induced CAC is associated with the expression of pro-inflammatory and anti-inflammatory cytokines, such as TNF-α, IFN-γ, IL-1β, IL-6, IL-4, and IL-10 [22,23]. As shown in Figure 3a-f, mRNA levels of pro-inflammatory cytokines (TNF-α, IFN-γ, IL-1β, and IL-6) were increased in the CON group, whereas those of anti-inflammatory cytokines (IL-4 and IL-10) were decreased. However, these changes in mRNA levels were reversed by the administration of 5-ASA and CJ (Figure 3a–f). To confirm these effects, we evaluated the levels of TNF-α, IFN-γ, IL-1β, IL-6, IL-4, and IL-10 in a serum by ELISA. Consistent with the mRNA results, the CON group showed increased TNF-α, IFN-γ, IL-1β, and IL-6 levels and decreased IL-4 and IL-10 levels (Figure 3g–l). The increase/decrease in cytokine levels was reversed in the PC and CJ groups, indicating that CJ modulates the expression of pro-inflammatory and anti-inflammatory cytokines in mice with AOM/DSS-induced CAC.

### 3.4. CJ Suppresses Activation of NF-κB Signaling in AOM/DSS-Induced CAC Mice

**Figure 4:**
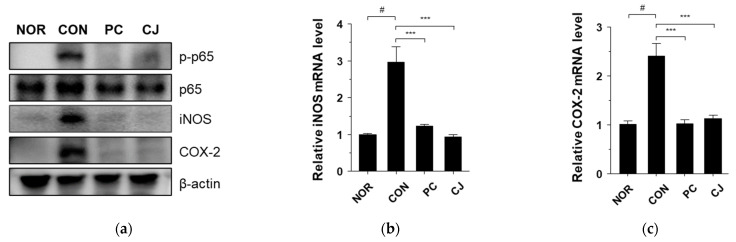
Effects of CJ on AOM/DSS-induced CAC-associated signaling pathways: (**a**) protein levels of phosphorylated p65, p65, iNOS, and COX2; and mRNA levels of (**b**) iNOS and (**c**) COX-2. Values are means ± standard deviation (*n* = 8); NOR, normal group; CON, control group; PC, positive control group; CJ, cheonggukjang treated group. #, *p* < 0.05 versus normal group; ***, *p* < 0.001 versus control group.

Nuclear factor-κB (NF-κB) is involved in inflammatory responses, proliferation, differentiation, and apoptosis. Moreover, the activation of NF-κB contributes to IBD and tumor development [24,25]. In a previous study, we showed that the NF-κB signaling pathway is activated by DSS and suppressed by CJ in DSS-treated mice [13]. Therefore, we investigated whether CJ suppresses NF-κB activation in mice with AOM/DSS-induced CAC. The CON group showed a significant increase in phospho-p65 NF-κB expression, which was decreased in the PC and CJ groups (Figure 4a). NF-κB activation induced several inflammatory enzymes, such as inducible nitric oxide synthase (iNOS) and cyclooxygenase (COX-2) [26]. Therefore, western blotting and quantitative real-time PCR (qRT-PCR) were performed to evaluate the iNOS and COX-2 levels in the treated mice. The protein levels of iNOS and COX-2 were increased in the CON group (Figure 4a). However, 5-ASA and CJ administration inhibited iNOS and COX-2 expression (Figure 4a). These findings were confirmed by the mRNA levels of iNOS and COX-2 measured by qRT-PCR (Figure 4b,c). These findings indicate that CJ suppresses the phospho-NF-κB, iNOS, and COX-2 expression in mice with AOM/DSS-induced CAC.

### 3.5. CJ Improves Intestinal Integrity in Mice with AOM/DSS-Induced CAC

The colonic mucus layer and tight junctions act as a layered defensive barrier in the colon and play a vital role in regulating mucosal permeability to ions, nutrients, and water [27,28]. The mucus layer, protected by the secreted mucin protein MUC2, is in contact with the epithelial cells lining the intestine and is joined via tight junctions [29]. Tight junctions consist of transmembrane proteins such as occludin, claudin, and junctional adhesion molecules [30]. Occludin directly interacts with zonula occludens-1 (ZO-1) and regulates paracellular permeability [31]. The loss of the colonic mucus layer is responsible for various intestinal diseases, including IBD and CRC [32,33].

**Figure 5:**
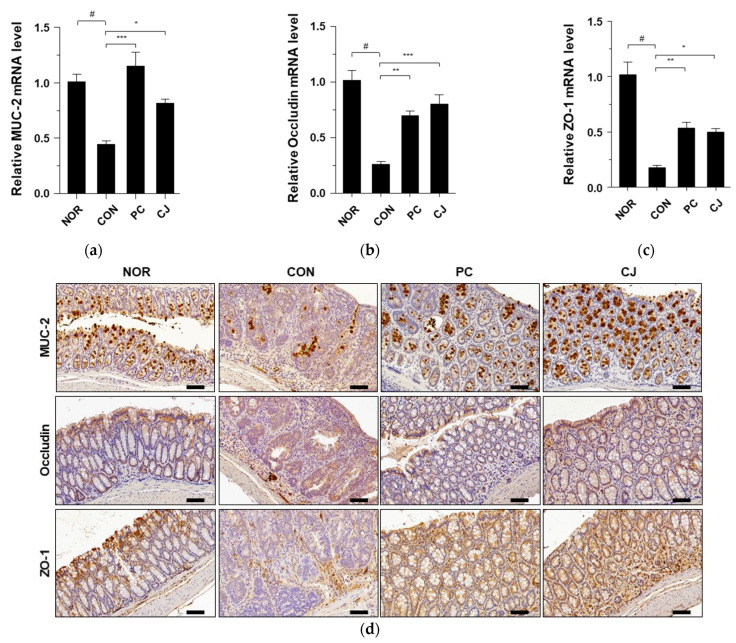
Effects of CJ on the expression of mucin-associated protein and tight junction proteins in mice with AOM/DSS-induced CAC. Shown are the mRNA levels of (**a**) Muc2, (**b**) occludin, and (**c**) ZO-1 in the different groups of mice. (**d**) Representative images of the colonic tissue immunohistochemically stained with MUC2, occludin, and ZO-1. Magnification, 200 X; scale bar, 60 μm. Values are means ± standard deviation (*n* = 8); NOR, normal group; CON, control group; PC, positive control group; CJ, cheonggukjang treated group. #, *p* < 0.05 versus normal group; ***, *p* < 0.001; **, *p* < 0.005; *, *p* < 0.05 versus control group.

We investigated the levels of mucin-associated protein (MUC2) and tight junction structural proteins (occludin and ZO-1) in the treated mice to assess the effects of AOM/DSS treatment on the colonic mucus layer. The mRNA levels of MUC2, occludin, and ZO-1 were significantly decreased in the CON group (Figure 5a–c). However, the administration of 5-ASA and CJ reversed the effects of AOM/DSS and significantly increased the mRNA levels of these markers (Figure 5a–c). Immunohistochemical staining confirmed the reduced expression of MUC2, Occludin, and ZO-1 in the CON group (Figure 5d). However, the administration of 5-ASA and CJ reversed these effects and increased the levels of MUC2, occludin, and ZO-1. These findings indicate that CJ may improve intestinal integrity by inducing the expression of mucin-associated and tight junction proteins in mice with AOM/DSS-induced CAC.

### 3.6. CJ Suppresses Tumor Growth by Regulating Apoptosis and Proliferation

**Figure 6:**
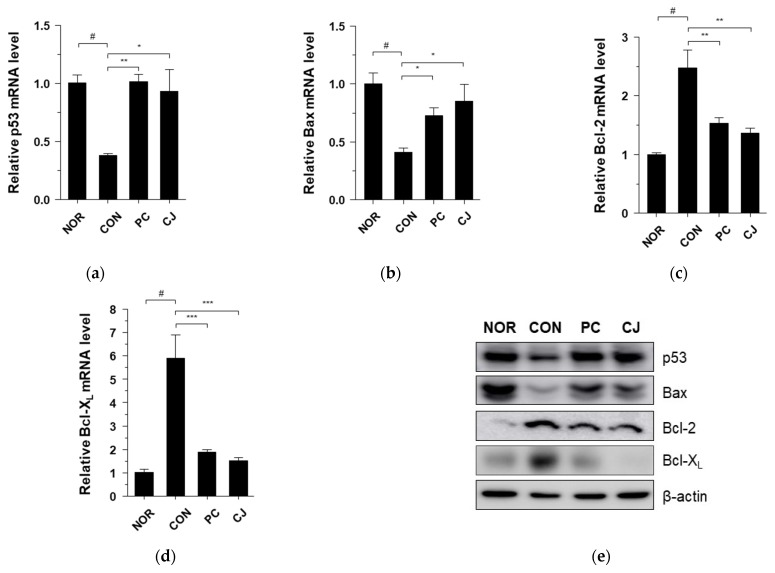

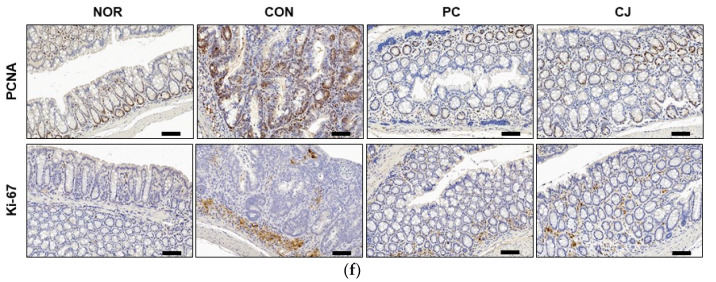
Effects of CJ on the expression of apoptosis and proliferation-related proteins in mice with DSS-induced colitis. mRNA levels of (**a**) p53, (**b**) Bax, (**c**) Bcl-2, and (**d**) Bcl-X_L_ in the different groups of mice were measured by qRT-PCR. (**e**) Protein levels of p53, Bax, Bcl-2, and Bcl-X_L_ were determined by western blotting. (**f**) Representative images of the colonic tissue immunohistochemically stained with PCNA and Ki67. Magnification, 200×; Scale bar, 60 μm. Values are means ± standard deviation (*n* = 8); NOR, normal group; CON, control group; PC, positive control group; CJ, cheonggukjang treated group. #, *p* < 0.05 versus normal group; ***, *p* < 0.001; **, *p* < 0.005; *, *p* < 0.05 versus control group.

The occurrence of CRC is closely associated with abnormal cell growth via the rapid proliferation and evasion of apoptosis [34]. The NF-κB signaling pathway regulates apoptosis and cell proliferation factors such as p53, Bax, Bcl-2, Bcl-X_L_, PCNA, and Ki67 [24,35]. In Figure 4a, we observed that the NF-κB signaling pathway was activated in the CON group. Thus, we evaluated the apoptosis and cell proliferation factors following treatment with AOM/DSS in this group. As shown in Figure 6a–d, the mRNA levels of pro-apoptotic markers, p53 and Bax, were significantly lower, and the mRNA levels of anti-apoptotic markers, Bcl-2 and Bcl-X_L_, were higher in the AOM/DSS-treated CON group compared with the NOR group. Treatment with 5-ASA and CJ reversed these effects (Figure 6a–d). Western blotting confirmed these mRNA results at the protein level (Figure 6e). Next, to evaluate the regulation of cell proliferation by AOM/DSS, cell proliferation markers were studied with immunohistochemical staining. PCNA and Ki-67 expression were higher in the AOM/DSS-treated CON group compared with the NOR group, while 5-ASA and CJ reversed this effect (Figure 6f). These findings indicate that CJ inhibits tumor growth by inducing apoptosis and suppressing the proliferation of tumor cells in mice with AOM/DSS-induced CAC.

## 4. Conclusions

This study demonstrates that CJ, made from the fermentation of boiled soybeans, significantly ameliorates AOM/DSS-induced pathological symptoms in mice including colonic shortening, spleen hypertrophy, tumor formation, and histopathological colonic changes, similar to the positive control (5-ASA). We also show that CJ and 5-ASA suppress the expression of pro-inflammatory cytokines and inflammatory mediators while increasing the expression of anti-inflammatory cytokines through the activation of NF-κB signaling pathways. Furthermore, CJ and 5-ASA attenuate tumorigenesis by inhibiting tumor growth through the induction of apoptosis and the inhibition of cell proliferation. These results suggest that CJ, which shows anti-cancer effects similar to 5-ASA, can be used as a functional food to prevent chronic inflammatory CRC.

## Figures and Tables

**Table 1:** Primer sequences.

Gene	Forward (5′-3′)	Reverse (5′-3′)
*TNF-* *α*	CTGAACTTCGGGGTGATCGG	GGCTTGTCACTCGAATTTTGAGA
*IFN-γ*	AGCCCTATTACAGCACAG	TTCTAACAACAAGTATCCC
*IL-6*	TGTCTATACCACTTCACAAGTCGGAG	GCACAACTCTTTTCTCATTTCCAC
*IL-1β*	CAACCAACAAGTGATATTCTCCATG	GATCCACACTCTCCAGCTGCA
*IL-4*	GGTCTCAACCCCCAGCTAGT	GCCGATGATCTCTCTCAAGTGAT
*IL-10*	CTTACTGACTGGCATGAGGATCA	GCAGCTCTAGGAGCATGTGG
*iNOS*	CGAAACGCTTCACTTCCAA	TGAGCCTATATTGCTGTGGCT
*COX-2*	TTTGGTCTGGTGCCTGGTC	CTGCTGGTTTGGAATAGTTGCTC
*p53*	CCCCTGTCATCTTTTGTCCCT	AGCTGGCAGAATAGCTTATTGAG
*Bcl-2*	GCTACCGTCGTGACTTCGC	CCCCACCGAACTCAAAGAAGG
*Bcl-X_L_*	GGCACTGTGCGTGGAAAGCGTA	CCGCCGTTCTCCTGGATCCA
*Bax*	AGACAGGGGCCTTTTTGCTAC	AATTCGCCGGAGACACTCG
*MUC-2*	ATGCCCACCTCCTCAAAGAC	GTAGTTTCCGTTGGAACAGTGAA
*Occludin*	TCTGCTTCATCGCTTCCTTAG	GTCGGGTTCACTCCCATTA
*ZO-1*	AGGACACCAAAGCATGTGAG	GGCATTCCTGCTGGTTACA
*GAPDH*	GACGGCCGCATCTTCTTGT	CAGTGCCAGCCTCGTCCCGTACAA

## Data Availability

Data is contained within the article.

## Abbreviation

CRC	Colorectal Cancer
IBD	Inflammatory Bowel Disease
CAC	Colitis-Associated Colorectal Cancer
CJ	Cheonggukjang
γ-PGA	poly γ-glutamic acid
DSS	Dextran Sulfate Sodium
AOM	Azoxymethane
NOR	Normal
CON	Control
PC	Positive Control
5-ASA	5-Aminosalicylic acid
qRT-PCR	Quantitative Real-Time PCR
H&E	Hematoxylin & Eosin
NF-κB	Nuclear Factor-κB
iNOS	Inducible Nitric Oxide Synthase
COX-2	Cyclooxygenase
